# The effect of augmentation of biochar and hydrochar in anaerobic digestion of a model substrate

**DOI:** 10.1016/j.biortech.2020.124494

**Published:** 2021-02

**Authors:** Jessica Quintana-Najera, A. John Blacker, Louise A. Fletcher, Andrew B. Ross

**Affiliations:** a School of Chemical and Process Engineering, University of Leeds, LS2 9JT Leeds, UK; b Institute of Process Research and Development, School of Chemistry, University of Leeds, LS2 9JT Leeds, UK; c School of Civil Engineering, University of Leeds, LS2 9JT Leeds, UK

**Keywords:** Anaerobic digestion, Biomethane, Biochar, Hydrochar, Surface functionality

## Abstract

The augmentation of biochar produced at 450 and 600–650 °C and hydrochar produced at 250 °C has been investigated using biochemical methane potential experiments of cellulose. The feedstocks used for the char production included the lignocellulosic (oak wood), macroalgae (*Fucus serratus*) and aquatic plant (water hyacinth). Biomethane production was improved with the addition of lower-temperature biochars from oak wood (285 mL CH_4_/g VS) and water hyacinth (294 mL CH_4_/g VS), corresponding to 7 and 11% more than the control. The addition of these two biochars increased the methane production rate of 2.4 and 2.3 times the control, respectively. Higher temperature biochars showed no difference. Conversely, all hydrochars and macroalgae biochars augmentation reduced methane generation by 57–86 %. The chemical and structural composition of each of the chars differed significantly. Surface oxygen functionality appears to be the most important property of the biochars that improved digestion performance.

## Highlights

• Low-temperature biochars doubled biomethane production rate and enhanced yields.
• Seaweed biochars and hydrochars from various feedstocks reduced biomethane yields.
• Biochars with high levels of oxygen surface groups favoured biomethane augmentation.

## Graphical abstract

**Figure:**
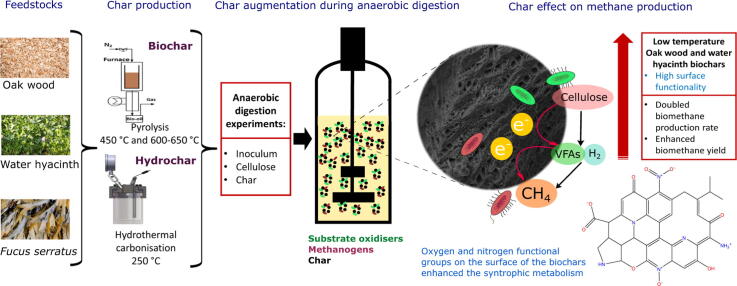

## 1 Introduction

Anaerobic digestion (AD) is a dual purpose technology designed for the sustainable management of waste and the production of renewable energy in the form of biogas. However, AD often suffers operational instability due to the occurrence of inhibitory compounds either within the original biomass or produced during its conversion. Several strategies have been proposed for alleviating AD imbalance while improving methane yields and production rates, including the use of additives. Recently, the addition of carbonaceous materials during AD, such as activated carbon, graphene, carbon nanotubes, biochar (BC) and hydrochar (HC) has gained attention (Zhang et al., 2018).

BC is produced by pyrolysis under anoxic or limited oxygen conditions, whereas HC is produced by hydrothermal carbonisation (HTC) within hot compressed liquid water at elevated autogenous pressure. The advantages of using chars, BC and HC, over other adsorbent carbon materials include: their low cost, the potential to use a wide range of biomass feedstocks for their production, their environmental sustainability, improvement of the digestate, and them having advantageous physicochemical properties that can be further tailored to fulfil desired characteristics (Lee et al., 2017).

Even though the use of chars has been directed mostly towards soil amendment and energy storage, publications of char addition during AD have reported positive benefits. Including mitigation of ammonium inhibition (Mumme et al., 2014), promotion of archaea growth, increase in methanogenic microbial metabolism, and a reduction of microbial lag time (Shanmugam et al., 2018). HC has also been considered a source of nutrients for AD and a promoter of hydrolysis and acidification reactions (Codignole Luz et al., 2018, Mumme et al., 2014). Hence, due to the physicochemical and structural properties of the chars, their potential application as adsorbents or catalyst supports is gaining attention (González et al., 2013).

The chars have heterogeneous structures with attractive characteristics, such as inertness, high availability of functional groups, and particularly for BC, high surface area (SA) and pore volume (PV) (González et al., 2013, Downie et al., 2009). Oxygenated functional groups (OFGs) on the surface are reported to serve as anchoring and interaction sites for biomolecules, whether they be whole cells, ions or inhibitory compounds (Oliveira-Castro et al., 2017). A well-developed porous structure is also attractive for providing a larger surface of interaction for the immobilisation and protection of cells and biofilm formation (Ramírez-Montoya et al., 2015). The addition of chars has reported to provide a buffering effect for the intermediary organic acids of AD (Zhang et al., 2018). Given the importance of pH for achieving a syntrophic balance, pH adjustment is often necessary for counterbalancing the variations resulting from organic acids generation. The above represents a challenge and cost, especially at large scale and rural communities that generate electricity and gas with rudimentary digestors. Hence, it is attractive to assess char augmentation as an option for maintaining a balanced pH and replacing pH adjustment.

Functional groups promote syntrophic metabolism by facilitating direct interspecies electron transfer (DIET) interactions between H_2_-producing bacteria and H_2_-consuming methanogens (Zhao et al., 2015). The latter arises when conductive materials, such as BCs, replace the role of conductive-pili and/or c-type cytochrome appendages found on the outer cells, responsible of the DIET interactions (Wu et al., 2020). The BCs catalyse the reductive reactions by facilitating the transfer of electrons from bulk chemical electron donors to a receiving organic compound, enhancing thus reaction rates and kinetic efficiencies. OFGs within the redox-active structures quinone-hydroquinone moieties and/or conjugated π-electron systems within the chars aromatic sub-structures are responsible for the DIET interactions (Klüpfel et al., 2014, Zhang et al., 2010).

Given the nature of the thermochemical processes, the physical, chemical and functional properties of BC and HC differ significantly, broadening the portfolio of potential applications. Several studies have investigated the influence of the feedstock and processing conditions on the final properties of the chars (Zhao et al., 2013). To date, a detailed investigation of the properties of BC and HC and their effect on AD has not been reported. Hence, an understanding of the physicochemical characteristics of the chars is of great importance for their successful development as adsorptive material additives during AD. In addition, the publications of BC addition in AD have employed principally BC from woody biomass. For this work, feedstocks with largely different composition and origin were selected for BC and HC production, including oak wood (OW), water hyacinth (WH) and *Fucus serratus* (FS). To the best of our knowledge, no information is available specifically regarding the pyrolysis of FS and WH and the further use of the BCs for AD amendment.

Overall, this research aims to develop a better understanding of how the augmentation of BC and HC can influence AD operation. Therefore, the objectives of the present work are to: (i) Produce a set of well characterised BCs and HCs from a diverse range of different feedstocks; (ii) investigate the influence of feedstock and processing conditions on the chemical and physicochemical properties of the chars, (iii) investigate the impact of BC and HC augmentation to conventional single-stage mesophilic anaerobic digestion of a model substrate.

## 2 Materials and methods

### 2.1 Biochar and hydrochar production

The OW-BCs were obtained from a commercial pyrolysis plant at 450 and 650 °C operated by Proininso (Spain), designated as OW-BC450 and OW-BC650, respectively. WH was collected from Lake Victoria, (Uganda) and provided by The Centre for Research in Energy and Energy Conservation (CREEC), Uganda, whereas FS was collected from the shores of Aberystwyth, Wales, UK and provided by Aberystwyth University. The BCs from WH (WH-BC450 and WH-BC600) and FS (FS-BC450 and FS-BC600) were produced by slow pyrolysis at 450 and 600 °C for 1 h. The HC samples from the three feedstocks were obtained by HTC at 10% (w/v) biomass loading, and 250 °C for 1 h. Further details on experimental facilities used can be found elsewhere (Hammerton et al., 2018, Smith et al., 2018).

### 2.2 Characterisation of biochar and hydrochar properties

Proximate analysis of the chars was determined using a thermogravimetric analyser (TGA) Mettler Toledo (TGA/DSC 1). Elemental analysis was performed with an automatic CHNS Thermo Instruments Flash EA 1112 Series and expressed as a percentage of total dry weight, with total oxygen (O) determined by difference. The pH measurement followed the methodology reported by Singh et al. (2017). SA and PV were quantified by gas adsorption, the chars were first outgassed in a vacuum oven by heating stepwise 50 °C every 30 min until reaching 200 or 150 °C for BC and HC, respectively and held for 1.5 h. Immediately before analysis, the chars were further outgassed by a continuous N_2_ flux at room temperature. A Tristar 3000 Micromeritics was used for the analysis, with two adsorptive gases, N_2_ at 77 K and CO_2_ at 273 K. The non-local density functional theory (NLDFT) models for infinite slit carbonaceous materials for CO_2_ at 273 K and N_2_ at 77 K were selected for the analysis of pores with 0.35–1.0 and 0.35–100 nm, respectively (Tarazona, 1985, Tarazona et al., 1987).

The spectroscopic analysis was performed by attenuated total reflectance Fourier transform infrared (ATR-FTIR) and X-ray photoelectron spectroscopy (XPS). FTIR was performed in an *iS10 Nicolet* ATR-FTIR spectrophotometer fitted with a diamond crystal. An average of 36–34 scans was taken over a range of 4000–400 cm^−1^, with a resolution of 4 cm^−1^, and reading collection every 5 min for background and sample subtraction. XPS measurements were acquired using a Specs system with high-intensity XR50 X-ray monochromatic Al Kα (1486.71 eV) source and a Phoibos 150 hemispherical electron analyser. The analysis of BCs took place at ultrahigh vacuum 10^-9^ Pa, the survey XPS spectra were acquired with a pass energy of 25 eV, in steps of 0.1 eV and dwell time of 0.1 s. High-resolution C 1 s, O 1 s and N 1 s spectra were obtained with a pass energy of 3 eV. Whereas for HCs, the XPS measurement was acquired using an EnviroESCA system near ambient pressure (NAP), under an argon atmosphere at a gas flow of 2 mL/min. The HCs XPS spectra for the survey and the high-resolution spectra were obtained with a pass energy of 100 and 50 eV, respectively. The Tougaard Background and CASA XPS software were used for curve fitting.

### 2.3 Source and preparation of Inoculum

The anaerobic inoculum was collected from the mesophilic wastewater treatment plant Esholt in Bradford, United Kingdom, and stored at 4 °C until use. Before use, the inoculum was homogenised by passing it through a mesh (1 mm). The total solids (TS) and volatile solids (VS) were quantified gravimetrically according to the APHA (2005).

### 2.4 Determination of biomethane potential

Biochemical methane potential (BMP) measurement was performed using an Automatic Methane Potential Test System (AMPTS II) (Bioprocess Control, Sweden). The reactors consisted of 500 mL standard bottles with a working space of 400 mL, inoculum 5 g VS/L and cellulose 5 g VS/L. Char loading 3% (w/v) was selected based on preliminary BMP tests conducted with different chars at 1–5% (w/v). Some cases showed better performance at 1% and others at 5%, however, they all show similar improvements with 3% in comparison to the control (data not shown). The reactors were flushed with nitrogen for assuring anaerobic conditions, incubated at 37 °C for 28 days and automatically stirred for 60 s every 10 min. A control filled only with inoculum and cellulose and a blank containing only inoculum were run in parallel to act as a reference BMP and to determine residual BMP emissions.

The experimental BMP values were fitted to the modified Gompertz model (Equation (1)) according to Zwietering et al. (1990), using SPSS Statistics 26 software.

(1) $$\mathrm{BMP}\left(t\right)={\mathrm{BMP}}_{\max}∙exp\left\{-exp\left[\frac{\mu_{m}∙e}{{\mathrm{BMP}}_{\max}}\left(\lambda -t\right)+1\right]\right\}$$

where; BMP(t) = Cumulative methane yield (mL CH_4_/g VS) at time t (day), BMP_max_ = Maximum methane yield (mL CH_4_/g VS), µ_m_ = Methane production rate (mL CH_4_/g VS·day), λ = Lag phase (days), e = exp(1).

This experiment aimed to evaluate BC and HC produced from very different feedstocks on AD. Principally their addition on AD performance, including methane generation, kinetic parameters, volatile fatty acids (VFAs) and changes in pH. A model substrate, cellulose, was selected for assuring that variations in these parameters are attributed exclusively to the chars.

### 2.5 Analysis of volatile fatty acids by gas chromatography.

To evaluate the effect of the chars on the accumulation of AD by-products, VFAs analysis was performed with an Agilent 7890A gas chromatograph, a DB-FFAP column (30 m × 0.32 mm, film thickness of 0.5 µm) and a flame ionisation detector (FID) at 200 °C with nitrogen as make-up gas. An autosampler injected 10 µL of the sample at a 5:1 split ratio with the inlet port operated at 150 °C and the carrier gas helium at 10 mL/min. The column oven program started at 60 °C and held for 4 min, then increased to 140 °C with a ramp of 10 °C/min. Afterwards, the temperature raised to 200 °C with a ramp of 40 °C/min and held for 5 min. The comparative standards used were a volatile acid standard mix (Supelco) and alcohols made from high purity single reagents. Data was acquired with ChemStation software.

### 2.6 Statistical analysis

BMP runs were conducted in triplicate except for the FS-BCs and HCs systems that were performed in duplicate, with the average values reported. The Shapiro-Wilks test showed normality (p > 0.05) for the BMP data. Comparison of the BMP for the BCs and HCs were made by an analysis of variance (ANOVA), whereas the individual effect for each BC, and interactions was determined by *Tukey post-hoc* test. These same BMP values were compared to the control without char with a *t*-test, all at a confidence level of p < 0.05 using the SPSS Statistics 26 software.

## 3 Results and discussion

### 3.1 Characterisation of the chars

#### 3.1.1 Selection of feedstocks

The feedstocks selected for this work were chosen based on the diversity of their origin and composition. The growth environments for OW, FS and WH are forestry, marine, and freshwater, respectively. First, OW is recalcitrant lignocellulosic biomass composed mainly of cellulose (44%), hemicellulose (24%) and lignin (24%), with negligible protein content (Andérez Fernández et al., 2018). Second, WH is an aquatic perennial free-floating invasive plant, rich in C, N and ash, with average crude fibre content (46%), crude protein (18%), and considerable cellulose and hemicellulose content (Ye, 2017). Third, FS is a brown seaweed composed up to 55% of carbohydrates (primarily laminarin and mannitol) and rich in ash (Jung et al., 2013).

The chemical composition of the three untreated feedstocks is listed in Table 1. The main differences were observed for inorganic, O and N content since these values were considerably lower for OW than for WH and FS. This is highly relevant when considering the positive effect of ash in AD regarding nutrient imbalance and alkalinity and its surface functionality on adsorptive properties (Amonette and Joseph, 2009). Thus, the differences in biochemical composition of the origin feedstocks impact the properties of their resulting BCs and HCs, and thus their application during AD.

**Table 1:** Chemical composition, surface area and porosity of the biochars and hydrochars.

Feedstock	Char	N(%)	C(%)	H(%)	O(%)	S(%)	VM(%)	FC(%)	Ash(%)	pH	S_CO2_(m^2^/g)	V_o, CO2_(cm^3^/g)	S_N2_(m^2^/g)	V_o, N2_(cm^3^/g)
	Raw	1.5	50.8	7.4	37.4	ND	72.5	24.7	2.9	–	–	–	–	–
Oak wood	OW-BC450	0.6	65.7	2.7	19.3	ND	21.1	67.2	11.7	9.9	221	0.08	3.4	0.05
	OW-BC650	0.8	76.5	1.4	7.0	ND	11.8	73.9	14.3	9.3	237	0.09	2.4	0.05
	OW-HC250	1.2	62.0	5.0	29.4	ND	57.9	38.5	3.82	3.9	44	0.01	0.4	0.01
	Raw	2.7	38.7	3.4	40.6	0.1	85.4	0.0	14.6	–	–	–	–	–
Water hyacinth	WH-BC450	1.4	26.1	1.2	54.7	0.2	32.8	43.9	23.4	9.1	39	0.02	5.0	0.05
	WH-BC600	2.2	38.7	0.8	21.0	0.0	26.9	35.9	37.3	10.6	90	0.03	5.3	0.05
	WH-HC250	3.3	50.1	4.2	20.9	0.1	56.4	23.0	20.6	5.6	37	0.01	1.3	0.02
Fucus serratus	Raw	1.4	44.2	6.1	32.5	1.6	74.5	11.3	14.2	–	–	–	–	–
	FS-BC450	2.4	39.2	1.8	21.6	1.7	48.4	18.8	33.3	11.1	72	0.02	ND	ND
	FS-BC600	2.7	40.4	0.9	21.5	0.3	36.1	29.3	34.6	12.1	64	0.02	0.3	0.01
	FS-HC250	2.4	54.9	4.6	22.7	1.1	64.1	21.6	14.3	6.2	41	0.10	6.8	0.08

VM volatile matter, FC fixed carbon, ND not detected, - not measured, S_CO2_ surface area and V_o,CO2_ pore volume obtained with CO_2_ adsorption at 273 K, S_N2_ surface area and V_o,N2_ pore volume obtained with _N2_ adsorption at 77 K; O content was determined by subtracting from 100% the C, N, H, S and ash; proximate composition is expressed in dry based content.

#### 3.1.2 Chemical composition of the biochars

BC and HC structure, composition and functionality are governed by both the nature of the given biomass feedstock and the processing conditions used for their production, particularly temperature (Mafu et al., 2017). The significance of the thermochemical process can be observed in Table 1, which compares the chemical composition of the BCs and HCs produced from each feedstock under different temperature/process conditions. Among the different feedstocks, the OW-BCs exhibited the highest reduction of volatile matter (VM) and lowest O, N and ash content. Conversely, the biochars from both WH and FS showed a lower reduction of VM and higher levels of ash, O and N content.

Increasing the pyrolysis temperature from 450 to 600–650 °C significantly reduced the VM and O-content, while increased FC, due to a more developed aromatisation. Such is the case for the OW-BCs, since the degradation of their carbohydrate fraction occurs in a stepwise manner below 600 °C, whereas at higher temperatures, a further conjugation of aromatic bonds predominates (Mafu et al., 2017). Similarly, the WH-BCs exhibited a large degree of carbonisation as illustrated by a drastic increase in fixed carbon (FC) and ash content. Conversely, the FS-BCs exhibited different VM and FC, although similar composition, based on CHNS and ash content, in accordance with its pyrolytic behaviour as previously reported (Anastasakis et al., 2011). The compositional changes between the untreated feedstocks and their corresponding BCs are the consequence of the volatilisation of organic matter, reduction of OH and CH_3_ functionality and an increase in C = C, due to an increase in carbon content and aromaticity (Amonette and Joseph, 2009). The loss of organic matter also leads to the concentration of inorganics and N in the chars. Thus, BCs produced from the same feedstock, but different conditions often have a very different composition.

#### 3.1.3 Chemical composition of the hydrochars

Table 1 lists the properties of HCs produced at 250 °C and demonstrates that HCs had different properties to BCs with significantly higher VM, H and N, but lower pH and ash content. The C-content of HCs was typically higher than the lower temperature (450 °C) BCs, however, they contain similar levels of O-content. Besides, HCs are generally considered to have higher functionality than BCs as a consequence of the higher levels of humin formed during the HTC process (Sumerskii et al., 2010). The VM content of the three HCs covered a range of 56 to 64%, although the net loss of VM varied among the HCs probably due to the differences on their complex carbohydrate structure and particularly for OW the recalcitrance of the lignin fraction (Ross et al., 2008). The HCs also showed an increase of C and loss of O and H-content mainly due to dehydration, deoxygenation and decarboxylation reactions occurring during HTC.

The HCs from the aquatic biomasses, WH and FS, exhibited a higher inorganic content than the terrestrial OW-HC as stated in previous reports (Brown et al., 2020, Gao et al., 2013, Smith et al., 2018). However, the levels of ash in OW and FS HCs remained similar to the origin biomass, whereas the ash content of WH-HC is increased from 14.6 to 20.6%. The changes in inorganic content during HTC are highly dependent on the composition of the feedstock. For instance, alkali metals such as Na and K are easily removed into the process waters, whereas the removal of P and alkaline earth metals (Mg and Ca) is more limited (Smith et al., 2016a). Despite the selective extraction of inorganic components into the process waters during HTC, the overall level of inorganics within the HC can still increase. Such was the case for WH-HC, which is likely a combination of large VM loss resulting in a concentration of inorganics and higher levels of soluble inorganic components (Ca and Si) (Ye, 2017). All HCs were produced under identical conditions; thus their compositional changes could be attributed to the inherent biochemical composition of each feedstock.

#### 3.1.4 Surface area and porosity measurements

The N_2_ and CO_2_ adsorption isotherms for the BCs and HCs are shown in the supplementary material. The shape of the N_2_ isotherms indicated several limitations, such as weak adsorbent-adsorbate interactions, condensation of the gas into the meso and macropores, and the inability of N_2_ to access the narrow microporosity of the chars. Even though N_2_ adsorption at 77 K is the most commonly used technique for SA analysis, its implementation on BCs and HCs may not be ideal. Because of this, the adsorptive CO_2_ at 273 K was also prefered since CO_2_ molecules present larger kinetic energy, improved ability to reach narrow pores, presenting less diffusional difficulties than N_2_ at 77 K. The shape of the CO_2_ isotherms demonstrated the heterogeneity of these materials by indicating the presence of a wide range of pore sizes (Lozano-Castelló et al., 2004, Thommes et al., 2015).

The SA and PV of BCs and HCs obtained from both N_2_ and CO_2_ adsorption are summarised in Table 1. For the N_2_ at 77 K, the SA of the chars was considerably lower (2.28–18.35 m^2^/g), due to the mentioned limitations. In contrast, the SA of BCs and HCs by CO_2_ at 273 K adsorption was found in a range 39–237 and 37–58 m^2^/g, respectively. The SA of BCs was influenced principally by the pyrolysis temperature, followed by the feedstock composition (Zhao et al., 2013). The OW-BCs exhibited the larger SA, probably due to the recalcitrant lignin fraction that partially preserved the original structure during pyrolysis. Unlike OW, the BCs from WH and FS possessed higher ash and VM content which reflected in a lower degree of carbonisation, less developed porosity and smaller SA (Downie et al., 2009). The HCs, on the other hand, exhibited similar SA despite the feedstock, although significantly lower than the BCs.

#### 3.1.5 Fourier transform infrared spectroscopy

The interferograms from BC and HC originated from the same parent feedstock followed a similar behaviour as observed in the supplementary material. All the chars exhibited stretching bands for OH (3650–3200 cm^−1^) which indicated the presence of alcohols, phenols and carboxylic acids, aliphatic v(CH) from –CH_2_ groups (2950–2920 cm^−1^), C≡C (2260–2150 cm^−1^), C = C (2050–2000 cm^−1^), aryl groups (phenolic groups) (1715–1695 cm^−1^), COO^–^ (1610–1550 cm^−1^) and CO_3_^-2^ (1426–1410 cm^−1^). In addition to those structures, HCs presented a wider range of groups including aliphatic v(CH) from CH_3_ groups (2870–2840 cm^−1^), open-chain amides (1570–1515 cm^−1^), ether groups (1275–1200 cm^−1^) and polysaccharide region (1160–1020 cm^−1^) (Fleming and Williams, 1966, Johnston, 2017, Peterson et al., 2013). All the chars exhibited aliphatic, OFGs and aryl groups, although the HCs spectra were generally more intense than BCs, indicating a broader surface functionality.

#### 3.1.6 X-ray photoelectron spectroscopy

The semi-quantification of the main components of the XPS full survey and the high-resolution C 1 s, O 1 s and N 1 s spectra are outlined in Table 2. For the survey, there was no consistent trend for the C and O content towards increasing the pyrolysis temperature, whereas N content appeared to reduce. On the other hand, the HCs exhibited a considerable N content, particularly large for FS-HC250, but lower O values than the BCs, however, the HCs were analysed under different conditions (NAP). The chars also exhibited significant amounts of inorganics, including Na, Cl, Ca, Si and K, principally for WH and FS, due to their higher ash content. While the full survey spectrum provided a general breakdown of the surface chemistry of the chars, the detailed analysis of their surface functionality was obtained from the high-resolution C 1 s, O 1 s and N 1 s spectra.

**Table 2:** Atomic ratios composition of biochar and hydrochar obtained by X-ray photoelectron spectroscopy main survey and high resolution C 1 s, O 1 s and N 1 s spectra.

Char	Survey			C 1 s					O 1 s		N 1 s			
	C 1 s(%)	O 1 s(%)	N 1 s(%)	C–C(%)	C-O(%)	C = O(%)	COO (%)	π-π* (%)	O = C (%)	O-C(%)	N-6(%)	N-5(%)	N-Q(%)	N-X(%)
OW-HC250	70.4	14.1	3.9	31.4	34.1	19.5	15.0		16.3	83.7	17.8	59.1	9.8	13.4
OW-BC450	81.1	16.0	1.2	23.5	22.1	35.9	2.6	15.9	10.8	89.2	21.0	61.3	9.6	8.1
OW-BC650	78.6	17.0	0.9	24.1	38.8	37.1			34.1	65.9	47.6	30.4	13.1	8.9
WH-HC250	79.1	9.7	2.8	15.1	38.9	39.8	6.2		16.7	83.3	32.4	11.1	28.6	27.6
WH-BC450	58.0	21.2	3.3	22.5	49.9	11.1	16.4		3.5	96.5	33.9	43.7	10.0	10.0
WH-BC600	67.3	18.9	1.5	45.0	31.5	15.8	7.8		67.6	32.4	41.8	33.8	16.5	8.0
FS-HC250	71.3	10.1	14.4	25.6	52.6	15.5	6.3		23.1	76.9	34.2	28.2	12.6	25.0
FS-BC450	61.6	18.6	1.9	14.4	51.1	30.5	4.0		70.2	29.8	16.0	50.8	20.0	15.2
FS-BC600	43.2	28.5	0.2	26.4	47.6	15.3	10.8		79.2	20.8	27.4	28.5	21.4	22.7

**Survey XPS spectra**: C 1 s (284 eV), O 1 s (532 eV) and N 1 s (399 eV); **C 1 s XPS spectra**: aromatic C–C (284.4–285.2 eV), C-O (286.1–286.6 eV), C = O (287.6–288.2 eV), COO (288.8–289.3 eV) and π-π* (291.3 eV); **O 1 s XPS spectra:** O = C (531.3–532.3 eV) and O-C (533.3–533.9 eV); **N 1 s XPS spectra:** pyridinic-N (N-6) (397.5–398.9 eV), pyrrolic-N (N-5) (399.0–400.4 eV), quaternary-N (N-Q) (4015–401.8 eV) and N-oxide (N-X) (403.9–404.1 eV) (Konno, 2016, Watts and Wolstenholme, 2003).

The C 1 s XPS spectra deconvolution of the BCs indicated a raise in aromatic C–C at higher pyrolysis temperature, accompanied by a large variability of OFGs, and particularly for OW-BC450 the presence of π-π* transition due to conjugation from the aromatic structure. The C 1 s of HCs exhibited a lower content of C–C and a greater proportion of OFGs, principally C-O groups. When analysing changes in OFGs due to temperature increase, the O 1 s spectra provided a better understanding (Smith et al., 2016b). Accordingly, the O 1 s showed principally O-C groups for most low-temperature BCs and HCs. The content of the O = C groups within the BCs was greater for those produced at higher temperatures. The N 1 s spectra showed pyrrolic-N (N-5) and pyridinic-N (N-6) as the dominant nitrogen functional groups (NFGs) on the surface of the chars. The N-5 content was lower for the higher-temperature BCs since N-5 is reported to transform into N-6 and quaternary-N (N-Q) with the raise of pyrolysis temperature due to condensation reactions. HCs, on the other hand, showed a considerable content of N-oxide (N-X) and N-Q in agreement with previous reports (Pels et al., 1995). Conclusively, the XPS analysis indicated the presence of OFGs and NFGs for all chars, although at different extents.

#### 3.1.7 Summary of biochar and hydrochar properties

The characterisation of BCs and HCs exhibited differences in physicochemical properties as a function of temperature/processing conditions and feedstocks selection. The main differences between the BCs from the three feedstocks were the significantly higher SA and lower VM, N, O and ash content for OW-BCs in comparison to the BCs from WH and FS. The seaweed FS-BCs also showed a higher S-content and alkalinity. On the other hand, the HCs had lower SA, pH and ash content, but higher VM, O and N than the BCs. The surface chemistry of the chars was proportionally related to their bulk chemical composition. Thus, those with greater O, N and ash content also showed higher OFGs, NFGs and inorganics on their surface.

### 3.2 Biochemical methane potential with biochar and hydrochar augmentation

#### 3.2.1 Effect of biochar and hydrochar on BMP

**Fig. 1:**
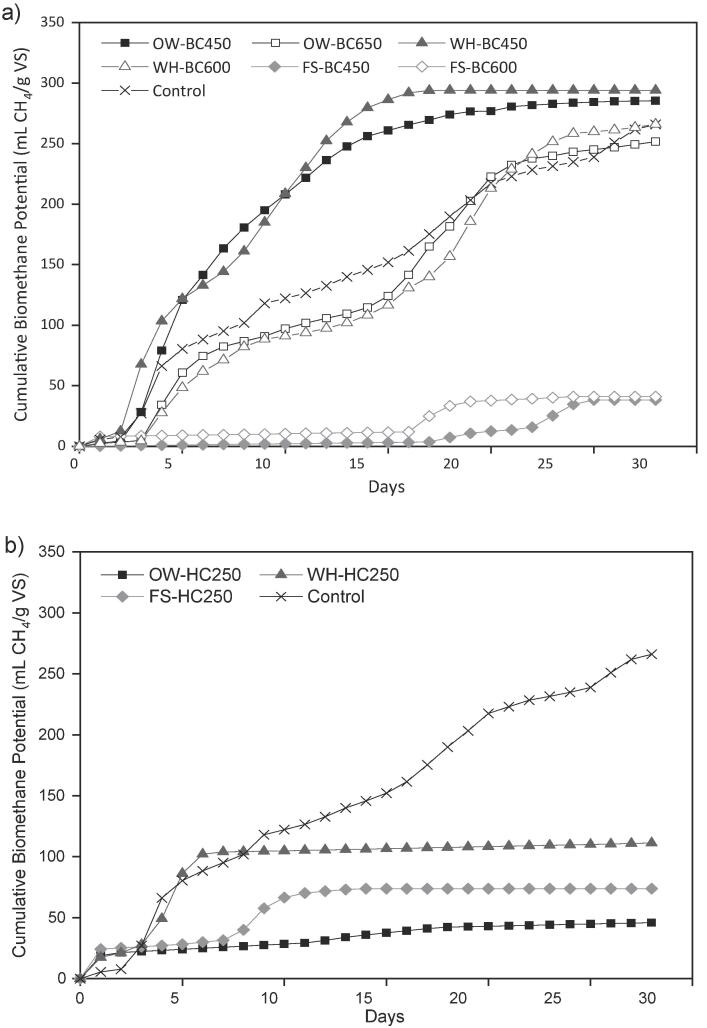
Biochemical methane potential during anaerobic digestion of cellulose augmented with chars produced from oak wood (OW), water hyacinth (WH), *Fucus serratus* (FS) and non-char control: a) biochar and b) hydrochar. BC450 biochar produced at 450 °C; BC600 biochar produced at 600 °C; BC650 biochar produced at 650 °C; HC250 hydrochar produced at 250 °C.

The BMP curves with and without the addition of the different BCs are shown in Fig. 1a. There was a lag phase of 2–3 days for most systems before they began to produce methane, except for FS-BCs whose initial production extended until the 17th day. The BMP of most systems was found near the mid-exponential by the 7th day. Though, OW-BC450 and WH-BC450 exhibited a faster production rate by reaching the steady-state earlier than the remaining systems. The final BMP for the experiments augmented with OW-BC450 (285 ± 12 mL CH_4_/g VS) and WH-BC450 (294 ± 8 mL CH_4_/g VS) showed a significant difference over the no BC control (266 ± 3 mL CH_4_/g VS) (p < 0.05), which corresponded to a 7 and 11% improvement, respectively. Conversely, the addition of OW-BC650 and WH-BC600 resulted in 5% less production than the control, though considerably less than the highest producers. Further, Fig. 1a shows that the addition of both FS-BCs drastically affected methane generation, with almost 85% less final BMP than the control. Similarly, Qin et al. (2020) added BC from different feedstocks to the AD of glucose and reported different BMP yields for each BC. The woody BCs improved the yields, which they attributed to their high SA, electron-donating capacity (EDC) and predominance of methanogenic archaea. Whereas the corn stalk BC showed a low SA, drastically reduced BMP and led to a decreased of *Methanosarcina*.

These findings indicated that, while BCs had a range of characteristics that may improve AD, not all the BCs are the same and some BCs may be better suited for AD. The FS-BCs were not suitable for enhancing digestion behaviour. This could be partially attributed to the toxic or inhibitory by-products originated by the pyrolysis of seaweed, including phenols, N-nitrosodimethylamine, 5-methyl-furfural, among others (Ross et al., 2009). Even though there was no significant difference in BMP between the systems augmented with OW-BC450 and WH-BC450 (p < 0.05), their addition was the most successful having improved methanogenesis compared to the control.

The Influence of HC augmentation on BMP behaviour is shown in Fig. 1b. For HC, no initial lag phase was observed, and an initial methane yield was generated on day 1. This may be due to the presence of VFAs and sugars adsorbed onto the surface of the HCs. After this, initial burst, a secondary lag phase occurred before there was a recovery and methane was generated, although at significantly lower levels (58–83%) than the control. The WH-HC was the least inhibitory with 111 mL CH_4_/g VS. The maximum BMP with the addition of FS-HC and OW-HC were 70 mL and 40 mL CH_4_/g VS, respectively. This greater level of inhibition increased for high lignin-containing feedstocks such as OW. Reports HC and HC-slurries addition to AD resulted in increased methane yields (Brown et al., 2020, Codignole Luz et al., 2018, Wang et al., 2017). However, most studies aimed to use the HC as a substrate and showed that as the temperature of HTC increases, the HC became more inhibitory. Even though lower-temperature HCT had not been assessed in this study, HCT at 180–200 °C may result in HC with reduced levels of inhibitory compounds.

The effect of HC on AD is attributed principally to the humic acids largely present in the HC and undesirable by-products. The humic acids can act as electron acceptors improving the acetic acid production, while further competing for them with the methanogens, reducing thus VFAs consumption and methane generation (Wang et al., 2017). During HTC, Maillard reactions produce N-containing compounds (melanoidins and N-heterocycles), considerably inhibitory for fermentative bacteria (Lin et al., 2018). The acidic nature and the high levels of phenolic functionality (FTIR) could also have affected the BMP production. Hence, the addition of these three different HCs resulted in low methane yields and could be regarded to inhibit methanogenesis.

#### 3.2.2 Kinetic assessment

The modified Gompertz model was employed to evaluate biomethane production (Table 3). The different chars added to the digester led to different kinetic parameters. The highest BMP_max_ of 321 mL CH_4_/g VS was achieved by WH-BC600, whereas WH-BC450 and the OW-BCs obtained similar values to the control. The highest µ_m_ was observed for OW-BC450 (28 mL CH_4_/g VS·day) and WH-BC450 (27 mL CH_4_/g VS·day), which represented 2.4 and 2.3 times the obtained with the control, respectively. OW-BC650 and WH-BC600 exhibited similar µ_m_ than the control, whereas FS-BCs and HCs reached considerably lower BMP_max_ and µ_m_. Most systems exhibited low λ values, suggesting that BC addition did not reduce λ. The parameter most affected by char addition is µ_m_ as shown for OW-BC450 and WH-BC450, which resulted in the most significant enhance.

**Table 3:** Biomethane experimental yield and kinetic parameters calculated with the modified Gompertz model during the augmentation of biochar and hydrochar in anaerobic digestion.

System	BMP_Exp_(mL CH_4_/g VS added)	BMP_max_(mL CH_4_/g VS added)	µ_m_(mL CH_4_/g VS·day)	λ(days)	R^2^
OW-HC250	45.85	68.78	1.22	0	0.946
OW-BC450	285.46	285.69	28.08	1.46	0.993
OW-BC650	251.63	262.45	13.06	2.60	0.972
WH-HC250	111.20	110.41	24.38	1.51	0.973
WH-BC450	294.15	298.39	27.31	1.18	0.988
WH-BC600	266.03	321.28	12.32	3.30	0.979
FS-HC250	73.85	75.79	6.31	0	0.930
FS-BC450	38.25	41.64	4.88	17.33	0.973
FS-BC600	41.05	56.24	1.90	3.63	0.884
Control	265.93	284.85	11.81	0.20	0.985

BMP_Exp_ maximum experimental methane yield, BMP_max_ maximum theoretical methane yield, µ_m_ methane production rate, λ lag phase (days), R^2^ coefficient of determination.

#### 3.2.3 Volatile fatty acids and pH

**Fig. 2:**
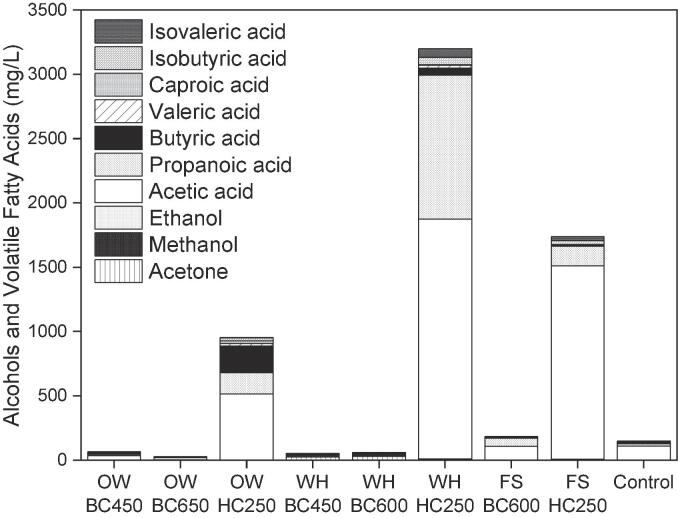
Alcohols and volatile fatty acids accumulated at the end of the anaerobic digestion augmented with biochar and hydrochar produced from oak wood (OW), water hyacinth (WH), *Fucus serratus* (FS) and non-char control.BC450 biochar produced at 450 °C; BC600 biochar produced at 600 °C; BC650 biochar produced at 650 °C; HC250 hydrochar produced at 250 °C.

The alcohols and VFAs accumulated at the end of the AD experiments augmented with the different BCs and HCs as shown in Fig. 2. Those systems that produced higher amounts of methane also resulted in a lower accumulation of VFAs. Both OW-BCs and WH-BCs reduced the accumulated VFAs to less than half the control. Conversely, the addition of all HCs resulted in a considerably higher accumulation of VFAs, in agreement with previous reports that used HC produced under similar conditions (Brown et al., 2020, Wang et al., 2017).

**Fig. 3:**
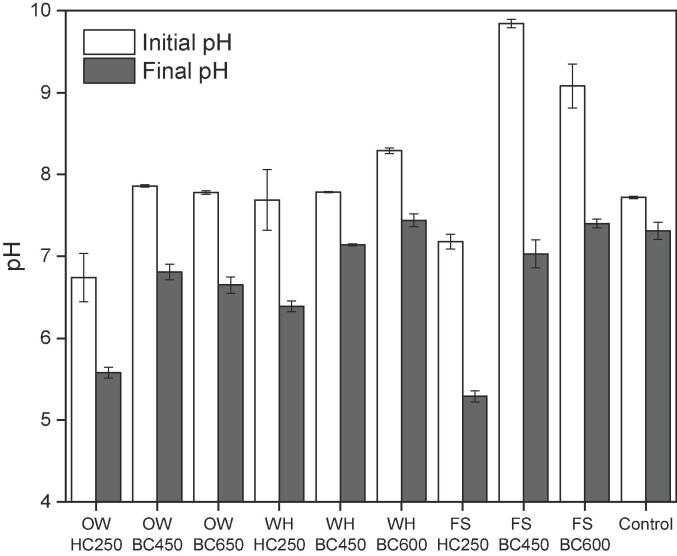
pH at the beginning and end of the anaerobic digestion augmented with biochar and hydrochar produced from oak wood (OW), water hyacinth (WH), *Fucus serratus* (FS) and non-char control.BC450 biochar produced at 450 °C; BC600 biochar produced at 600 °C; BC650 biochar produced at 650 °C; HC250 hydrochar produced at 250 °C.

To evaluate the effect of the chars on the pH of the digester, no pH adjustment was performed. The pH was measured once the chars were added and before the AD started and also at the end of the AD (Fig. 3). BCs are known to be more alkaline due to the enhancement of inorganics in the chars after pyrolysis, whereas HCs are well known to be acidic as listed in Table 1. Accordingly, all systems augmented with BC showed an initial alkaline pH, whereas the pH for the systems augmented with HCs and the control were closer to neutrality. Nevertheless, most systems started at a suitable pH since the optimal value for single stage AD is pH 6.8–7.4. However, the pH for the FS-BCs systems was particularly high (pH ~ 9), possibly due to its increased N-content and increased levels of alkali and alkaline earth metals. Initial alkaline conditions facilitate the hydrolysis of carbohydrates. However, pH as alkaline as the observed for FS-BCs could have hindered further VFA consumption and methanogenic activity.

At the end of the BMP experiment, the FS-BCs and HC systems suffered the greatest pH variations, followed by OW-BCs, WH-BCs and the control at a minor extent. Drastic pH changes often affect microbial communities, such as methanogens since pH below 6.5 and above 8.0 do not favour methane production. Despite the pH variations, the final pH for all BCs systems remained close to the optimal range. Conversely, the HC systems reached pH levels unfavourable for methanogens (pH 5.3–6.4) in agreement to the considerable higher VFAs accumulation. Given the pH variations, it is not possible to attribute a buffering effect from either BC or HC.

#### 3.2.4 Effect of biochar augmentation

In this work, it has been demonstrated that lower temperature BCs enhanced the methane production rate and yield during AD. Thus, developing an understanding of how the BCs properties affect their fitness for augmentation is crucial. This knowledge could result advantageous since these given properties can be tailored by controlling the pyrolysis conditions and feedstock selection (Lee et al., 2017).

Given the large differences between the lower temperature BCs from OW and WH, it is important to understand which inherent physicochemical properties could have influenced their positive impact in AD. Both BCs showed comparable alkaline pH and VM. OW-BC450 showed low ash content, high SA (S_CO2_ = 221 m^2^/g) (Table 1) and PV (0.08 cm^3^/g). Conversely, WH-BC450 exhibited a considerably higher ash content, low SA (S_CO2_ = 39 m^2^/g) and PV (0.02 cm^3^/g). The high SA and porosity of OW-BC450 could have provided the support and environment for the interaction and/or adsorption of microorganisms. This benefit could have been reduced for WH-BC450, given its lower SA. While reports of BCs with low and high SA exhibited the same positive effect on AD since often the higher SA is due to not accessible nanoporosity (Cruz Viggi et al., 2017). The inorganics within the BCs could provide a source of alkalinity and trace nutrients, especially for WH-BC450 given its considerably higher ash content. The soluble inorganics (Cl, Ca and K) could enhance the BC conductivity and subsequently improve DIET interactions (Chintala et al., 2014). Nevertheless, the redox properties of the BCs are originated principally from their organic electron-accepting and donating moieties, and to a lesser extent from their inorganic constituents (Klüpfel et al., 2014).

The XPS analysis of both OW-BC450 and WH-BC450 showed a large contribution of OFGs, with a predominance of O-C, followed by O = C (Table 2). These groups could indicate hydroquinone and quinone moieties, respectively. Klüpfel et al. (2014) reported that BCs produced at 400–500 °C showed an extensive redox buffering capacity dominated by quinone/hydroquinone functionalities. Whilst increasing pyrolysis temperature reduced these groups. The above agrees with the behaviour observed for OW-BC650 and WH-BC600. These systems reached BMP yields close to the control, which suggests that no further functionality benefits were provided.

Similarly to the ultimate composition, the XPS main survey showed a higher N content for WH-BC450 (3.3%) than for OW-BC450 (1.2%) (Table 2). The XPS N 1 s spectra showed a variety of NFGs for all chars. The NFGs are reported to provide sites for adsorption for organic and inorganic compounds. NFGs can also contribute to the electrocatalytic potential for redox reactions. The above is the combined effect of N-6 and N-Q with an adjacent C-atom to redistribute the charge and subsequently promote the redox reaction (Leng et al., 2019). This property is more relevant for WH-BC450 since this BC showed a greater contribution of N-6 than OW-BC450. In summary, OW and WH low-temperature BCs could have favoured the intimate proximity between the substrate-oxidisers and the methanogens. The functionality and redox capacity of these BCs could have triggered syntrophic DIET interactions and enhance the methanogenic performance.

## 4 Conclusions

The present study demonstrates that low-temperature biochar can improve methane production during AD. Since the physicochemical characteristics of the biochars improving AD differed largely, their positive effect could be attributed to a summary of beneficial properties such as high surface area and OFGs for OW-BC450 or even a greater content of OFGs, NFGs and conductive inorganics for WH-BC450. Conversely, seaweed biochars and hydrochar supplementation negatively affected AD and promoted VFAs accumulation. The nature of the parent materials along with the thermochemical processing conditions influenced the char properties.

## CRediT authorship contribution statement

**Jessica Quintana-Najera:** Conceptualisation, Investigation, Data curation, Formal analysis, Methodology, Project administration, Resources, Software, Validation, Visualisation, Writing - original draft, Writing - review & editing. **A. John Blacker:** Conceptualisation, Investigation, Project administration, Resources, Supervision, Writing - review & editing. **Louise A. Fletcher:** Conceptualisation, Investigation, Project administration, Resources, Supervision, Writing - review & editing. **Andrew B. Ross:** Conceptualisation, Funding acquisition, Investigation, Project administration, Resources, Supervision, Validation, Visualisation, Writing - review & editing.

## Declaration of Competing Interest

The authors declare that they have no known competing financial interests or personal relationships that could have appeared to influence the work reported in this paper.
