# Supplementary material for: The effect of augmentation of biochar and hydrochar in anaerobic digestion of a model substrate
Source: Bioresour Technol. 2021 Feb;321:124494. doi: 10.1016/j.biortech.2020.124494 (PMC7812375; doi:10.1016/j.biortech.2020.124494)
Supplement: Supplementary Data 1 [file mmc1.docx]

The effect of augmentation of biochar and hydrochar in anaerobic digestion of a model substrate

Jessica Quintana-Najera^a^, A. John Blacker^a,b^, Louise A. Fletcher^c^, and Andrew B. Ross^a,^^[[1]](#footnote-1)^*

^a^School of Chemical and Process Engineering, University of Leeds, LS2 9JT Leeds, UK

^b^Institute of Process Research and Development, School of Chemistry, University of Leeds, LS2 9JT Leeds, UK

^c^School of Civil Engineering, University of Leeds, LS2 9JT Leeds, UK

**Supplementary data**

**Fig. S1.** N_2_ at 77 K adsorption (●)/desorption (○) isotherms for biochar and hydrochar from a) oak wood (OW); b) water hyacinth (WH); and c) *Fucus serratus* (FS).

BC450 biochar produced at 450 °C; BC600 biochar produced at 600 °C; BC650 biochar produced at 650 °C; HC250 hydrochar produced at 250 °C. FS-BC450 showed no valid isotherm.

**Fig. S2**. CO_2_ at 273 K adsorption isotherms for biochar and hydrochar from oak wood (OW), water hyacinth (WH) and *Fucus serratus* (FS).

BC450 biochar produced at 450 °C; BC600 biochar produced at 600 °C; BC650 biochar produced at 650 °C; HC250 hydrochar produced at 250 °C.

**Fig. S3.** Attenuated total reflectance Fourier transform infrared (ATR-FTIR) spectra of selected biochar and hydrochar from oak wood (OW), water hyacinth (WH) and *Fucus serratus* (FS).

BC450 biochar produced at 450 °C; BC600 biochar produced at 600 °C; BC650 biochar produced at 650 °C; HC250 hydrochar produced at 250 °C.

**Fig. S4**. X-ray photoelectron spectroscopy (XPS) spectra for: a) biochar analysed at ultra-high vacuum; b) hydrochar analysed at near ambient pressure.

OW oak wood; WH water hyacinth; FS Fucus serratus; BC450 biochar produced at 450 °C; BC600 biochar produced at 600 °C; BC650 biochar produced at 650 °C; HC250 hydrochar produced at 250 °C. The peaks of Ar 2s and Ar 2p correspond to the argon flow used during the hydrochar XPS analysis.

1. *Corresponding author

   E-mail address: [a.b.ross@leeds.ac.uk](mailto:a.b.ross@leeds.ac.uk) (A. Ross) [↑](#footnote-ref-1)
